# Long-term-video monitoring EEG and 18F-FDG-PET are useful tools to detect residual disease activity in anti-LGI1-Abs encephalitis: A case report

**DOI:** 10.3389/fneur.2022.949240

**Published:** 2022-08-16

**Authors:** Sara Cornacchini, Antonio Farina, Margherita Contento, Valentina Berti, Martina Biggi, Alessandro Barilaro, Luca Massacesi, Valentina Damato, Eleonora Rosati

**Affiliations:** ^1^Department of Neurosciences Drugs and Child Health, University of Florence, Florence, Italy; ^2^Department of Neurology 2, Careggi University Hospital, Florence, Italy; ^3^Department of Neurology, Pordenone Hospital, Pordenone, Italy; ^4^Department of Biomedical Experimental and Clinical Sciences “Mario Serio”, Florence, Italy; ^5^Nuclear Medicine, Careggi University Hospital, Florence, Italy

**Keywords:** anti-LGI1-antibodies, autoimmune encephalitis, rituximab, 18F-FDG-PET, EEG

## Abstract

**Background:**

The use of CD20-depleting monoclonal antibodies has shown to improve the long-term outcome of patients with anti-leucine-rich glioma-inactivated protein 1 antibodies (anti-LGI1-Abs) encephalitis after first-line immunotherapy, but currently predictive markers of treatment response and disease activity are lacking.

**Case presentation:**

A 75-year-old man presented cognitive impairment and faciobrachial dystonic seizures (FBDS), with mild abnormalities at electroencephalography (EEG), normal brain magnetic resonance and cerebrospinal fluid (CSF) analysis. Anti-LGI1-Abs were detected in serum and CSF, and corticosteroids and intravenous immunoglobulins were administered. Despite partial cognitive improvement, 18F-fluoridesoxyglucose-positron emission tomography (18F-FDG-PET) showed the persistence of temporo-mesial hypermetabolism, and FBDS were still detected by long-term monitoring video EEG (LTMV EEG). Rituximab was therefore administered with FBDS disappearance, further cognitive improvement, and resolution of 18F-FDG-PET temporo-mesial hypermetabolism.

**Conclusions:**

Our experience supports the use of 18F-FDG-PET and LTMVEEG as useful tools to measure disease activity, evaluate treatment response and guide therapeutic decisions in the long-term management of anti-LGI1-antibody encephalitis.

## Introduction

Anti-leucine-rich glioma-inactivated protein 1 antibodies (Anti-LGI1-Abs) encephalitis is the second most common form of autoimmune encephalitis (1). Patients with anti-LGI1-Abs normally present with severe anterograde amnesia, psychiatric symptoms and seizures typically preceded by paroxysmal motor events known as faciobrachial dystonic seizures (FBDS), which are considered as pathognomonic for the disorder but are often unrecognized (2). Cerebrospinal fluid (CSF) analysis is often non-inflammatory, and brain magnetic resonance imaging (MRI) can be normal in a number of patients (2, 3). Early recognition of this condition could prompt the early start of immunotherapy resulting in a better outcome, especially of the cognitive impairment (4). Although improvement after first-line immunotherapy has been shown (2), long-term sequelae are present in about three quarter of cases (2, 3). Second-line therapies, especially rituximab, have proved to be effective in refractory anti-LGI1-Abs encephalitis (5, 6) and early use of anti-CD20 therapies could improve the long-term outcome of patients failing to recover from first-line immunotherapy (7), as seen in other conditions mediated by IgG4 antibodies (8). Currently, clinical and paraclinical biomarkers to predict the clinical course and monitor the response to treatment in autoimmune encephalitis are lacking. We herein describe a case of anti-LGI1-Abs encephalitis in which long-term-monitoring video electroencephalography (LTMV-EEG) and 18F-fluoridesoxyglucose-positron emission tomography (18F-FDG-PET) findings guided the long-term management.

## Case

**Figure 1:**
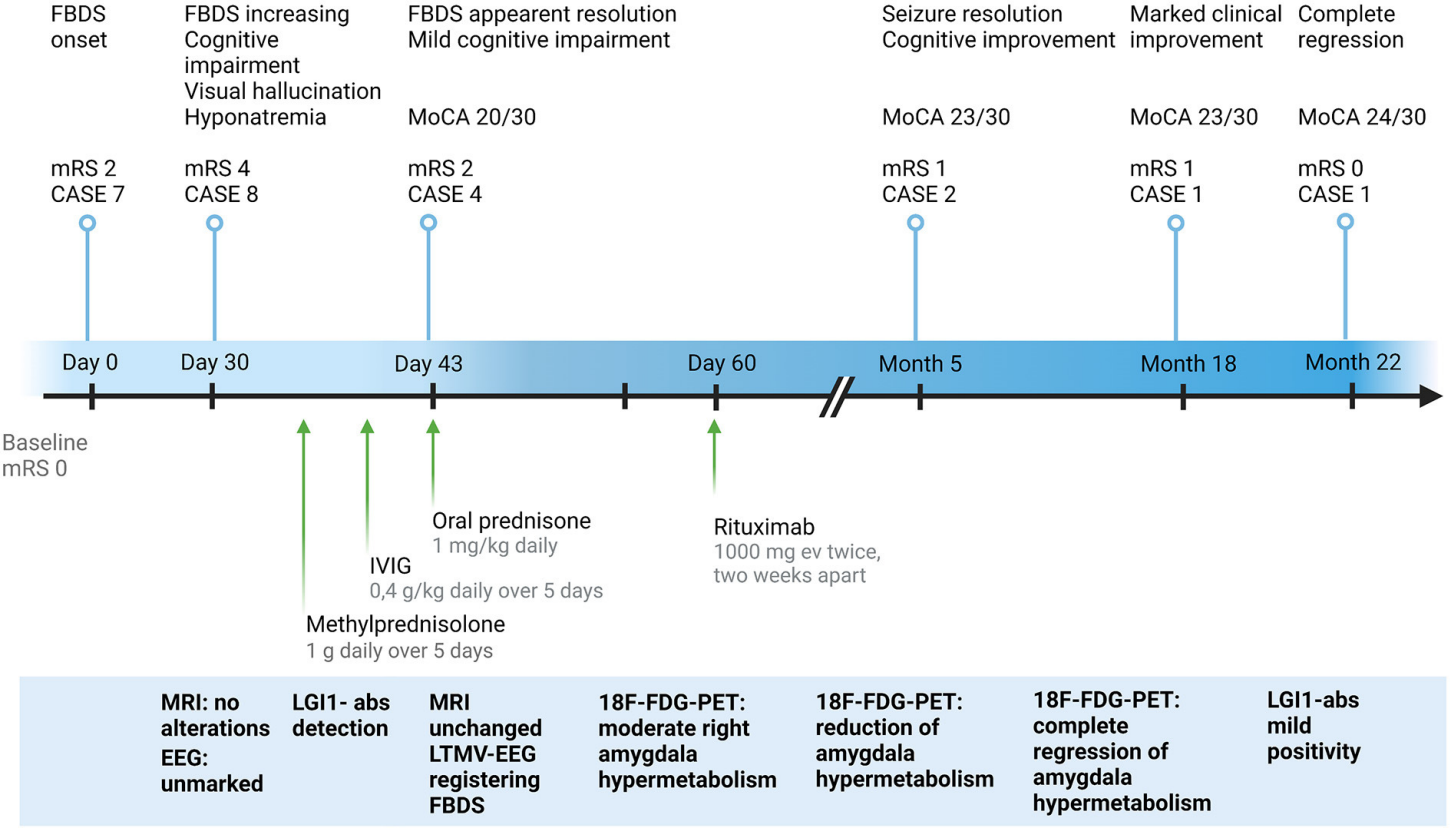
Clinical and paraclinical features of the study case from symptoms onset to last follow-up. CASE, Clinical Assessment Scale in autoimmune Encephalitis; EEG, electroencephalography; 18F-FDG-PET, 18F-fluoridesoxyglucose-positron emission tomography; FBDS, faciobrachial dystonic seizures; IVIG, intravenous immunoglobulins; LGI1-abs, leucine-rich glioma-inactivated protein 1 antibodies; LTMV-EEG, long-term-monitoring video electroencephalography; MoCA, Montreal Cognitive Assessment; MRI, magnetic resonance imaging; mRS, modified Rankin Scale. Created with BioRender.com.

**Figure 2:**
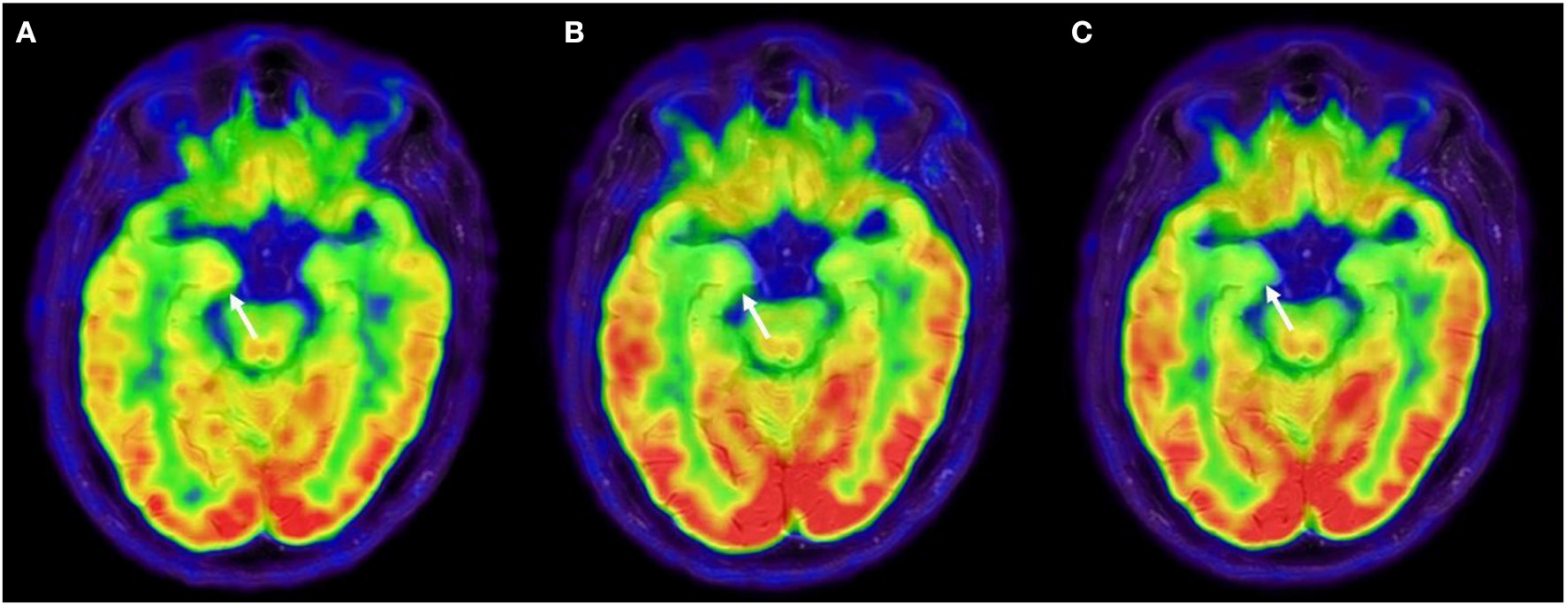
18F-FDG-PET evolution. **(A)** 18F**-**FDG-PET after first-line immune therapy showing moderate right amygdala hypermetabolism and bilateral temporomesial, frontoanterior and frontomesial hypometabolism, suggestive for persistence of disease activity. **(B)** 18F-FDG-PET was repeated after 3 months from rituximab, showing an attenuation of amygdala hypermetabolism and at 10 months from rituximab **(C)**, showing a complete regression of the amygdala hypermetabolism. 18F-FDG-PET, 18F-fluoridesoxyglucose-positron emission tomography.

A 75-year-old man presented with abrupt-onset of bilateral faciobrachial dystonic seizures (FBDS) (right > left), often accompanied by vocalization and oro-buccal automatisms. The patient was amnesic and unaware about these events. Patient past history reported only cutaneous psoriasis and benign prostatic hyperplasia. In few weeks, these episodes increased in frequency to up to 12–24 times a day and were associated by rare generalized tonic-clonic seizures, leading to patient's hospitalization (timeline of the clinical and paraclinical features are shown in Figure 1). At admission to the hospital, the patient presented memory disfunction, episodes of visual hallucinations and seizures leading to several falls [modified Rankin Scale (mRS) = 4, CASE score = 8]. Neurological examination did not reveal strength or sensory deficits, cranial nerves or coordination impairment. Brain MRI was unremarkable. EEG showed bilateral temporal slow waves without ictal activity. Blood tests revealed mild hyponatremia and positive anti-thyroglobulin and anti-thyroperoxidase antibodies. CSF analysis was normal. Anti-LGI1-antibodies were detected in serum and CSF by using commercial kits (Euroimmun), confirming the diagnosis of anti-LGI1-Abs encephalitis. Given the final diagnosis and the therapeutic failure of anti-seizure medications (ASM) with Levetiracetam and Lacosamide in the first month after disease onset, corticosteroids (intravenous methylprednisolone 1 g daily over 5 days followed by oral prednisone 1 mg/kg daily) and intravenous immunoglobulins (0.4 g/kg daily over 5 days) were administered leading to regression of hallucinations and apparent disappearance of seizure (mRS = 2, CASE score = 4). Nevertheless, LTMV-EEG revealed the persistence of paroxysmal events consistent with FBDS (around 25 in 86 h) and a distinctive behavioral pattern manifesting as “pedalage” during rapid eye movement (REM) sleep (Supplementary Video 1). Moreover, brain 18F-FDG-PET showed moderate right amygdala hypermetabolism and bilateral temporomesial, frontoanterior and frontomesial hypometabolism in spite of the normal brain MRI scan (Figure 2A). Lastly, neuropsychological evaluation was performed and showed moderate auditory-verbal memory loss [Montreal Cognitive Assessment (MoCA) 20/30]. Based on these findings—in the uncertainty of further clinical improvement—second-line therapy with rituximab (1,000 mg ev twice, 2 weeks apart) was administered with total regression of FBDS and further cognitive improvement (MoCA 23/30). PET scans showed progressive improvement of the alterations toward complete disappearance of the amygdala hypermetabolism up to 10-months after rituximab administration (Figures 2B,C). Oral corticosteroids were tapered to discontinuation along with ASM withdrawal. Twenty-two months after disease onset the patient was seizure-free, cognitive stable (MoCA 24/30) and independent in his daily activity (mRS = 0, CASE = 1). The patient and his caregivers reported complete remission of the disease and full return to previous daily life activities.

## Discussion

The identification of surface neural antibodies has improved the diagnosis of autoimmune encephalitis and the management of patients with this condition. Even though administration of second-line immunotherapy has been linked to a better prognosis (5, 9), second-line drugs as rituximab are normally reserved in patients with residual disease activity, due to the lack of reliable predictive biomarkers of clinical outcome (7). Moreover, a favorable outcome assessed by the clinical scales currently in use (e.g., mRS), can hide the persistence of cognitive deficits, as seen in other autoimmune encephalitis (10). The case presented here showed that the apparent clinical improvement after first-line immunotherapy was not sustained by LTMV-EEG monitoring, which documented the persistence of FBDS in the absence of severe cognitive decline and the presence of a possible REM sleep behavior disorder (11). This is consistent with previous studies that demonstrated the superiority of long-lasting EEG monitoring over short-lasting routine EEG in revealing subclinical and relapsing seizures (12, 13). We could not exclude that the patient could further improve over time even without escalation to second-line therapy, also based on the evidence that FBDS may require up to 3 months after initiation of immunotherapy to stop (4). However, we decided to administer rituximab based on its good safety profile (14), the evidence that cognitive impairment could be prevented by early termination of FBDS (4) and the persistent signs of inflammation at the 18F-FDG-PET. Importantly, while brain MRI scans remained unremarkable during the disease course, 18F-FDG-PET showed a better correlation with the clinical course, showing a reversible metabolic pattern, with a progressive attenuation of the amygdala hypermetabolism after rituximab treatment. The value of 18F-FDG-PET in detecting signs of limbic encephalitis in MRI-negative cases has been previously described (15, 16), supporting a diagnostic role of 18F-FDG-PET in the setting of autoimmune encephalitis (17). Here, 18F-FFG-PET has been used as an instrument to measure disease activity, leading to early second-line therapy, and then monitor treatment response. Because this is a single case report, and this is the principal limitation, further studies are required to investigate the cost/benefit ratio of a systematic evaluation of unconventional paraclinical tools to this purpose.

## Conclusion

In conclusion, our experience supports the use of paraclinical tools as FDG-PET and LTMV-EEG to monitor the disease activity in anti-LGI1-Abs encephalitis. Indeed, in previous studies the use of 18F-FDG-PET and LTMVEEG has been limited only to the diagnostic acute phase of disease. As clinical symptoms like mild cognitive impairment or FBDS can be unrecognized by patients and their care givers, clinicians should be aware that there are paraclinical tools that could detect residual disease activity for a condition susceptible to improvement after escalation to second-line therapy. Further prospective studies on large cohorts of patients are needed to evaluate the cost/benefit ratio of a systematic use of these instruments in this scenario.

## Data availability statement

The original contributions presented in the study are included in the article/Supplementary material, further inquiries can be directed to the corresponding author/s.

## Ethics statement

Ethical review and approval was not required for the study on human participants in accordance with the local legislation and institutional requirements. The patients/participants provided their written informed consent to participate in this study. Written informed consent was obtained from the individual(s), and minor(s)' legal guardian/next of kin, for the publication of any potentially identifiable images or data included in this article.

## Author contributions

SC and AF drafted the manuscript. MC, VB, and MB analyzed the EEG and PET data. VD and ER drafted and revised the manuscript for intellectual content. All authors contributed to the article and approved the submitted version.

## Conflict of interest

The authors declare that the research was conducted in the absence of any commercial or financial relationships that could be construed as a potential conflict of interest.

## Publisher's note

All claims expressed in this article are solely those of the authors and do not necessarily represent those of their affiliated organizations, or those of the publisher, the editors and the reviewers. Any product that may be evaluated in this article, or claim that may be made by its manufacturer, is not guaranteed or endorsed by the publisher.

## Supplementary material

Supplementary Video 1 LTMG-EEG with polygraphy. The recordings show paroxysmal events characterized by sudden brachial dystonic jerks of right arm, sometimes on either side, of few seconds in duration, sometimes with ipsilateral facial twitching. The episodes occurred both during wakefulness and, mostly during sleep without an ictal EEG correlate. The polygraphy recordings showed typical ocular movements of REM sleep (at EOG) followed by a distinctive behavioral pattern, manifesting as “pedalage” without any EEG ictal modification. LTMV-EEG, long-term-monitoring video electroencephalography; EOG, electrooculography; EMG-mylo, electromyography of the mylohyoid muscle; REM, rapid eye movements.
